# An example of a possible leech-bryozoan association in freshwater

**DOI:** 10.3897/zookeys.794.28088

**Published:** 2018-11-01

**Authors:** Anna L. Klass, Svetlana E. Sokolova, Alexander V. Kondakov, Yulia V. Bespalaya, Mikhail Yu. Gofarov, Alena A. Tomilova, Ilya V. Vikhrev, Ivan N. Bolotov

**Affiliations:** 1 Northern Arctic Federal University, Arkhangelsk, Russia; 2 N. Laverov Federal Center for Integrated Arctic Research, Russian Academy of Sciences, Arkhangelsk, Russia

**Keywords:** Bryozoa, bryozoan-associated epibionts, eastern Siberia, Glossiphoniidae, Hirudinea, Plumatellidae

## Abstract

Associations of various invertebrate species with bryozoans and sponges are a well-known marine phenomenon but such epizooic communities are far less diverse in freshwater environments. Here an occurrence of numerous leeches Alboglossiphoniacf.papillosa (Braun, 1805), in interstitial spaces between zooids of a colony of the freshwater bryozoan species Plumatellaaff.fungosa (Pallas, 1768) in Eastern Siberia is described. To the best of our knowledge, this record appears to be the first known example of a leech-bryozoan association, although such relationships deserve further research.

## Introduction

Marine ecosystems share numerous examples of commensal associations of various invertebrate taxa with sedentary animals such as bryozoans and sponges (Ristedt and Schuhmacher 1985, Wulff 2006). In contrast, the diversity of bryozoan- and sponge-associated epibionts in freshwater is much lower. For example, only a single freshwater shrimp-sponge association has been described so far (Rintelen et al. 2007), while multiple examples of such associations are known from the marine environment (Macdonald et al. 2006). Larvae of a few aquatic insects use freshwater sponges and bryozoans as a food source (Resh 1976, Ricciardi and Reiswig 1994), and several mites (Acari: Unionicolidae) occur in parasitic association with freshwater sponges (Edwards and Vidrine 2006). Freshwater bryozoans were found in association with an assemblage of filter feeders, including other bryozoans, sponges, hydroids, and caddisfly larvae (Ricciardi and Reiswig 1994). The interstitial spaces between zooids and around the edges of colonies may be occupied by several invertebrate taxa, i.e. tubicolous rotifers, ostracods, naidid oligochaetes, nematodes, and chironomids (Ricciardi and Reiswig 1994).

Here we describe an occurrence of numerous leeches, Alboglossiphoniacf.papillosa (Braun, 1805) inhabiting interstitial spaces between zooids of a colony of freshwater bryozoan species Plumatellaaff.fungosa (Pallas, 1768). We present the results of a molecular and morphological study of both the leech and the bryozoan species and briefly discuss possible explanations of this unusual finding. To the best of our knowledge, it represents the first documented case of a possible leech-bryozoan association.

## Materials and methods

**Figure 1.**
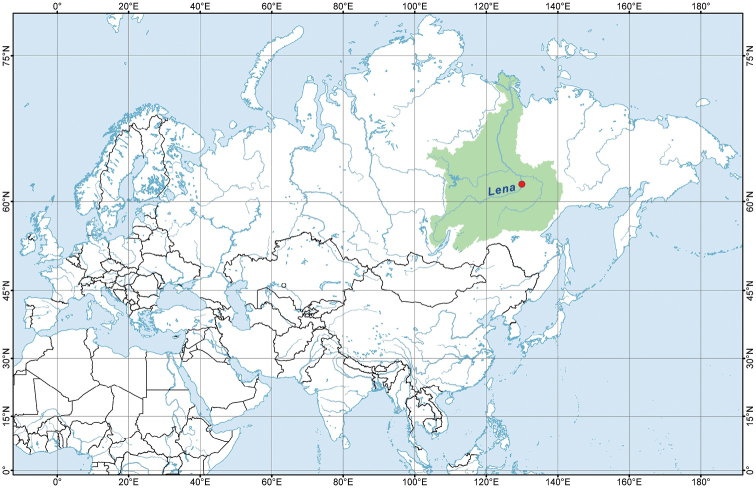
Map of Lena River basin, Eastern Siberia, with occurrence of the leech-bryozoan association in a floodplain lake (red dot).

A fragment of a willow branch with a bryozoan colony was collected by hand in a shallow coastal site of a floodplain lake in the Lena River basin, Yakutia, Eastern Siberia, Russia (Figure 1). This sample was preserved in 96% ethanol immediately after collecting. Leech specimens were collected and counted both from the ethanol solution in the vial and from interstitial spaces between the zooids on the basis of careful investigation of the colony under a stereomicroscope (Leica EZ 4D, Leica Microsystems, Germany). The total length of body in each leech specimen was measured using ocular micrometer of the same stereomicroscope.

Total genomic DNA was extracted from 96% ethanol-preserved tissue samples using the NucleoSpin Tissue Kit (Macherey-Nagel GmbH & Co. KG, Germany), following the manufacturer’s protocol. For molecular analyses we obtained partial sequences of the following markers: the mitochondrial *cytochrome c oxidase subunit I* gene (COI), the nuclear 18S ribosomal RNA (18S rRNA) and 28S ribosomal RNA (28S rRNA) genes. We amplified partial sequences of the COI and 18S rRNA genes for Alboglossiphoniacf.papillosa and partial sequences of the COI and 28S rRNA genes for Plumatellaaff.fungosa using standard primers (Tables 1, 2). The PCR mix contained approximately 200 ng of total cell DNA, 10 pmol of each primer, 200 μmol of each dNTP, 2.5 μl of PCR buffer (with 10 × 2 mmol MgCl_2_), 0.8 units Taq DNA polymerase (SibEnzyme Ltd.), and H_2_O was added for a final volume of 25 μl. Thermocycling was implemented with marker-specific PCR programs as follows: (i) COI: 95 °C (4 min), followed by 32 cycles at 94 °C (50 sec), 50 °C (50 sec), 72 °C (50 sec) and a final extension at 72 °C (5 min); (ii) 28S rRNA: 95 °C (4 min), followed by 22 cycles at 94 °C (50 sec), 60 °C (50 sec), 72°C (50 sec), and a final extension at 72 °C (5 min); (iii) *18S rRNA*: 95 °C (4 min), followed by 28–31 cycles at 94 °C (50 sec), 64 °C (50 sec), 72 °C (50 sec) and a final extension at 72 °C (5 min). Forward and reverse sequence reactions were performed directly on purified PCR products using an ABI PRISM® BigDye™ Terminator v. 3.1 reagents kit and run on an ABI PRISM® 3730 DNA analyzer (Thermo Fisher Scientific Inc., Waltham, MA, USA). The resulting sequences were checked by eye with BioEdit v. 7.2.5 (Hall 1999).

**Table 1.** List of sequenced specimens of Alboglossiphoniacf.papillosa and Plumatellaaff.fungosa from Eastern Siberia (a floodplain lake in the Lena River basin).

Species	Specimen Voucher*	*COI* acc. no.	*18S rRNA* acc. no.	*28S rRNA* acc. no.
Alboglossiphonia cf. papillosa	Hir13/1	MH286267	MH286273	n/a
	Hir13/2	MH286268	n/a	n/a
	Hir13/3	MH286269	n/a	n/a
	Hir13/4	MH286270	MH286274	n/a
	Hir13/5	MH286271	MH286275	n/a
Plumatella aff. fungosa	Por02	MH286272	n/a	MH286276

*Materials are deposited in the Russian Museum of Biodiversity Hotspots (RMBH), Institute of Biogeography and Genetic Resources, Federal Center for Integrated Arctic Research of the Russian Academy of Sciences (Arkhangelsk, Russia).

**Table 2.** Primer sequences for PCR amplification and sequencing.

Gene fragment	Primer’s name	Direction	Sequence (5'-3')	Reference
COI	LoboF1	Forward	kbtchacaaaycayaargayathgg	Lobo et al. (2013)
	LoboR1	Reverse	taaacytcwggrtgwccraaraayca	
18S rRNA	1F	Forward	tacctggttgatcctgccagtag	Giribet et al. (1996)
	4R	Reverse	gaattaccgcggctgctgg	
	3F	Forward	gttcgattccggagaggga	
	18Sbi	Reverse	gagtctcgttcgttatcgga	Whiting et al. (1997)
	18Sa2.0	Forward	atggttgcaaagctgaaac	
	9R	Reverse	gatccttccgcaggttcacctac	Giribet et al. (1996)
28S rRNA	D23F	Forward	gagagttcaagagtacgtg	Park and Ó’Foighil (2000)
	D2	Reverse	tccgtgtttcaagacgg	Jovelin and Justine (2001)

## Results

**Figure 2.**
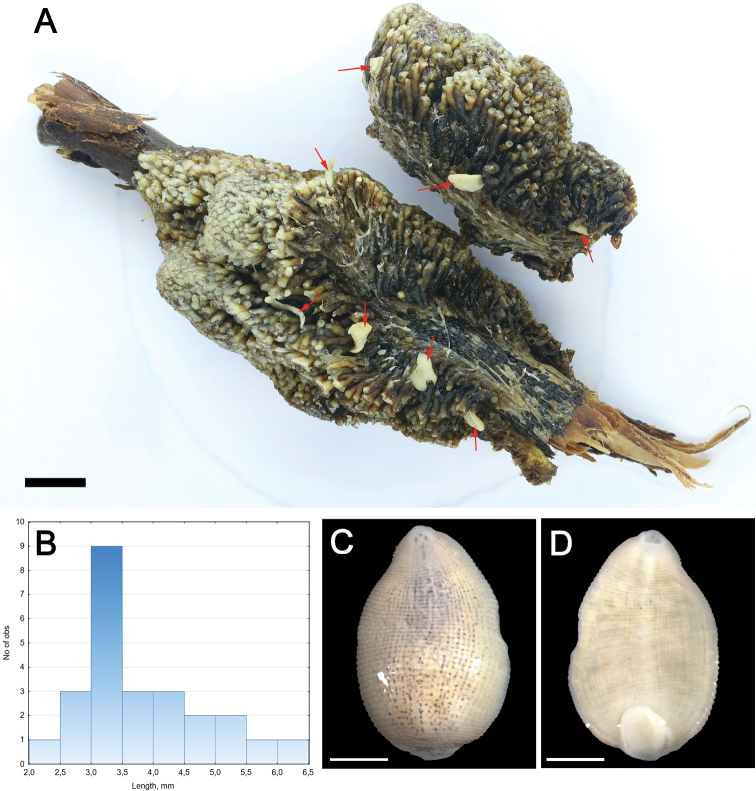
Leech-bryozoan association from a floodplain lake in Lena River basin, Yakutia, Eastern Siberia, Russia. **A** Leeches Alboglossiphoniacf.papillosa in interstitial spaces between zooids of a Plumatellaaff.fungosa colony (ethanol-preserved sample). The red arrows indicate leech specimens. **B** Size frequency histogram of the leech sample (*N* = 25). **C** Dorsal and **D** Ventral view of adult leech. Photographs Svetlana E. Sokolova. Scale bars: 5 mm (**A**); 1 mm (**C, D**).

A bryozoan colony (size of 40×20×25 mm) from Yakutia was heavily invaded by leeches. Twenty-five leeches (Figure 2A, C, D) were found in the interstitial spaces between zooids of this small colony. The living leeches were in spaces between zooids and were not visible externally when first collected (Figure 2A). The mean length of ethanol-preserved leeches (±s.e.m.) in the sample is 3.9±0.2 mm (min-max = 2.1–6.1 mm; *N* = 25). The sample includes 10 relatively large specimens of ≥4 mm long (40% of the total sample) (Figure 2B).

The results of molecular and morphological studies reveal that the leeches belong to Alboglossiphoniacf.papillosa (Braun, 1805), while the bryozoan species was identified as Plumatellaaff.fungosa (Pallas, 1768).

## Taxonomy

### Phylum Annelida Lamarck, 1809

#### Subclass Hirudinea Linnaeus, 1758

##### Family Glossiphoniidae Vaillant, 1890

###### Genus *Alboglossiphonia* Lukin, 1976

####### Alboglossiphonia cf. papillosa

Taxon classificationAnimaliaRhynchobdellidaGlossiphoniidae

(Braun, 1805)

######## Material examined.

RUSSIA: Eastern Siberia, Yakutia, a floodplain lake of the Lena River near the city of Yakutsk, 62.3076° N, 129.8999° E, 25 specimens from interstitial spaces between zooids of a Plumatellaaff.fungosa colony, Bolotov leg. (voucher no. RMBH: Hir13).

######## Comments.

Current taxonomy of this leech genus is uncertain. Closely related specimens of Alboglossiphoniacf.papillosa with homologous or very similar COI gene sequence (acc. nos. KM095100 and KM095101) (uncorrected *p*-distance <1%) were collected from the Gusinoye Lake, Yenisei Basin, Eastern Siberia (Kaygorodova et al. 2014). The BLAST search with the *18S rRNA* gene sequence returns the nearest members among the *Alboglossiphonia* taxa from the USA (acc. nos. AF115983 and AY962410) (Apakupakul et al. 1999; Siddall et al. 2005).

### Phylum Bryozoa Ehrenberg, 1831

#### Class Phylactolaemata Allman, 1856

##### Family Plumatellidae Allman, 1856

###### Genus *Plumatella* Lamarck, 1816

####### Plumatella aff. fungosa

Taxon classificationAnimaliaPlumatellidaPlumatellidae

(Pallas, 1768)

######## Material examined.

RUSSIA: Eastern Siberia, Yakutia, a floodplain lake of the Lena River near the city of Yakutsk, 62.3076° N, 129.8999° E, colony (size of 40×20×25 mm) on a willow branch fragment, Bolotov leg. (voucher no. RMBH: Por02).

######## Comments.

The species phylogenetically relates to *Plumatellafungosa* from Estonia (acc. no. KF805632) (Dash and Vasemägi 2014), but with a low similarity (uncorrected *p*-distance 3.8%). However, our specimen has 100% accordance with *Plumatellafungosa* from Austria (acc. no. KC462027) (Hartikainen et al. 2013) by the nuclear 28S rRNA gene sequence.

## Discussion

To the best of our knowledge, leeches have not previously been found in association with freshwater bryozoans, although the specific bryozoan colonial structure provides microscopic spaces between zooids supporting suitable microhabitats for diverse epizooic invertebrate communities (Ricciardi and Reiswig 1994, Bushnell and Rao 1979). Possible causes of leech aggregation within a bryozoan colony recorded by us are unclear. Possible explanations of this unusual finding could include: first, leeches may simply use bryozoans as a shelter. Second, they may also use zooids as a suitable food source, although there are no published observations of this. Third, leeches may be attracted by and feed on other animals inhabiting interstitial spaces between the zooids, e.g., oligochaetes and chironomids, although such animals were virtually absent within this colony. Another possible explanation is that the leeches concentrated within a bryozoan colony because they were recently hatched from the same parent located near this colony. However, the latter hypothesis seems to be unlikely, because Alboglossiphoniacf.papillosa is a small species, the length of which rarely extends 7–8 mm, and adult leeches of smaller size often occur (Lukin 1976). With respect to this, most of the leeches in our sample appear to be adults based on their size, and the shorter lengths of our ethanol-preserved specimens compared to the longer lengths of living leeches measured by Lukin (1976) can be accounted for by the fact that leeches contract in size when preserved.

Although this is the first recorded instance of a leech-bryozoan association, it may represent a largely overlooked phenomenon because of the hidden lifestyle of these small leeches. For example, the Japanese mussel leech *Batracobdellakasmiana* (Oka, 1910), which has a hidden life style within the mantle cavity of freshwater mussels, has only recently been discovered in the Russian Far East (Bolotov et al. 2017).

## Supplementary Material

XML Treatment for Alboglossiphonia cf. papillosa

XML Treatment for Plumatella aff. fungosa
